# Organotypic Cocultures of Human Pluripotent Stem Cell Derived-Neurons with Mammalian Inner Ear Hair Cells and Cochlear Nucleus Slices

**DOI:** 10.1155/2019/8419493

**Published:** 2019-11-20

**Authors:** Tomoko Hyakumura, Stuart McDougall, Sue Finch, Karina Needham, Mirella Dottori, Bryony A. Nayagam

**Affiliations:** ^1^Department of Audiology and Speech Pathology, The University of Melbourne, Parkville, Australia; ^2^The Bionics Institute, East Melbourne, Australia; ^3^The Florey Institute of Neuroscience and Mental Health, Parkville, Australia; ^4^Statistical Consulting Centre, The University of Melbourne, Parkville, Australia; ^5^Department of Surgery, The University of Melbourne, Parkville, Australia; ^6^Illawarra Health and Medical Research Institute, University of Wollongong, Australia

## Abstract

Stem cells have been touted as a source of potential replacement neurons for inner ear degeneration for almost two decades now; yet to date, there are few studies describing the use of human pluripotent stem cells (hPSCs) for this purpose. If stem cell therapies are to be used clinically, it is critical to validate the usefulness of hPSC lines *in vitro* and *in vivo*. Here, we present the first quantitative evidence that differentiated hPSC-derived neurons that innervate both the inner ear hair cells and cochlear nucleus neurons in coculture, with significantly more new synaptic contacts formed on target cell types. Nascent contacts between stem cells and hair cells were immunopositive for both synapsin I and VGLUT1, closely resembling expression of these puncta in endogenous postnatal auditory neurons and control cocultures. When hPSCs were cocultured with cochlear nucleus brainstem slice, significantly greater numbers of VGLUT1 puncta were observed in comparison to slice alone. New VGLUT1 puncta in cocultures with cochlear nucleus slice were not significantly different in size, only in quantity. This experimentation describes new coculture models for assessing auditory regeneration using well-characterised hPSC-derived neurons and highlights useful methods to quantify the extent of innervation on different cell types in the inner ear and brainstem.

## 1. Introduction

The hair cells and auditory neurons of the inner ear work cooperatively to convey acoustic information to the brain. Damage to either cell type is a primary cause of hearing loss. In addition, the synaptic connection between these two key sensory cell types is also vulnerable to damage and can result in permanent loss in transmission of acoustic information to the brain. Since damaged or degenerated auditory neurons do not spontaneously regenerate, potential approaches to ameliorate damage to sensory cell populations include stem cell replacement therapy. Specifically, neural stem cell therapy for hearing loss is aimed at restoring peripheral circuitry with the inner ear hair cells and also central circuitry with second-order neurons in the cochlear nucleus.

A number of studies have investigated *in vivo* stem cell replacement therapy for hearing loss over the past two decades, and these have been extensively reviewed in recent literature [1, 2]. Collectively, these studies have demonstrated that both mouse and human embryonic stem cells are capable of surviving within the host cochlea for an extended period of time without eliciting a severe host immune response [3–5]. Moreover, transplanted stem cell-derived neurons have been shown to innervate the sensory hair cells within deafened host cochlea [3, 4, 6], yet there is limited anatomical evidence of synaptogenesis on second-order neurons within the cochlear nucleus [4]. Importantly, improvements in hearing thresholds have been observed following stem cell implantation when compared to untreated deaf controls [4]. The observation that there is functional recovery of the deaf cochlea after stem cell therapy in these mammalian models suggests that some stem cells were able to successfully reconnect with both residual hair cells and with neurons in the auditory brainstem. Despite this improved functional recovery, only small numbers of new central synapses were discovered, and whilst nascent synaptic detection is understandably challenging *in vivo*, there is a paucity of quantitative evidence to detail the numbers and location of synapses made in these *in vivo* studies.

The two-dimensional nature of the *in vitro* environment confers several advantages over *in vivo* studies for the detection and quantification of neurite outgrowth and synaptogenesis. Whilst there are several published *in vitro* studies reporting new presynaptic terminals between inner ear hair cells and stem cell-derived neurons [7–11], there is a paucity of quantitative data to support the frequency of this stem cell synaptogenesis. In addition, there have been relatively few studies using human pluripotent stem cell (hPSC) lines for developing cell-based therapies for inner ear regeneration. In terms of investigating peripheral (hair cell) reinnervation, we [10, 11] and the others [7] have demonstrated that hPSCs differentiated toward a neurosensory lineage can extend neurites and make synapses on inner ear hair cells isolated from early postnatal rats [10, 11] and mice [7]. These nascent stem cell-derived synapses were immunopositive for the presynaptic markers synapsin I [10, 11] and synaptophysin [7]. Similarly, there are but two published studies describing central auditory reinnervation by human neural precursor cells (derived from a 9-week human embryo [12, 13]) and none describing the use of differentiated hPSC. If the described regenerative studies are to progress toward clinical translation, hPSCs warrant further interrogation in the auditory system.

In the present study, we report for the first time the growth of hPSC-derived sensory neurons toward postnatal inner ear hair cells and cochlear nucleus neurons in organotypic coculture with one and two other cell/tissue types. We quantify the extent of innervation into the peripheral (hair cell) and central (cochlear nucleus) target tissue *in vitro,* using a side-by-side comparison to relevant endogenous *in vitro* controls. The described models offer an excellent platform from which to interrogate the potential of different stem cell types (and indeed stages of differentiation of the same stem cell type) for cell transplantation purposes.

## 2. Materials and Methods

### 2.1. Animals

Time-mated, pregnant hooded Wistar rats were obtained from Laboratory Animal Services at the University of Adelaide. They were housed in standard conditions at the Biological Research Centre in the Department of Otolaryngology, University of Melbourne, Royal Victorian Eye and Ear Hospital. All procedures were conducted in accordance with the guidelines set by the Royal Victorian Eye and Ear Hospital Animal Research Ethics Committee (Approval number 11/235AR) and Australian code of practice for the care and use of animals for scientific purposes (7^th^ Edition, 2004, National Health and Medical Research Council of Australia).

### 2.2. Cell Lines

All experimentation using hPSC lines was approved by the University of Melbourne Human Ethics Committee (approval # 0605017) and conducted according to the National Health and Medical Research Council of Australia Guidelines for the Use of Human Stem Cells in Research (The National Statement, Chapter 2.1, 2007). The hPSC line H9 ([14], WA-09 (WiCell)) and human foreskin fibroblasts (CCD-1079Sk; ATCC) were used in this study.

### 2.3. Neural Differentiation of hPSCs

Human PSC lines were maintained and differentiated towards a neurosensory lineage as previously described [11, 15], and extensive molecular [16, 17] and physiological [16, 18] characterisation of these differentiated phenotypes supports their sensory phenotype. Briefly, undifferentiated hPSCs were maintained on mitomycin C-treated feeder layer, in Knockout Serum Replacement (KSR) medium (Dulbecco's modified Eagle's medium/nutrient mixture F-12 supplemented with 20% KSR, supplemented with 0.1 mM *β*-mercaptoethanol, 1% nonessential amino acids, 0.5% Penicillin-Streptomycin (all from Life Technologies), and 10-20 ng/ml basic fibroblast growth factor (PeproTech)), in an incubator set at 37°C and 5% CO_2_. Passaging was performed weekly.

The hPSCs were differentiated towards a neural lineage by first culturing in Neurobasal medium (Neurobasal A medium supplemented with 1% N-2 supplement, 2% B-27 supplement, 2 mM L-glutamine, and 0.5% Penicillin-Streptomycin (all from Life Technologies)) with 500 ng/ml of recombinant Noggin (R&D Systems) and 4 *μ*g/ml bFGF. Following 2 weeks of Noggin treatment, to induce neurosphere formation, Noggin-treated hPSC colonies were cut into small fragments and transferred into a low attachment 96-well plate (Corning) and cultured with Neurobasal medium supplemented with epidermal growth factor (EGF) and bFGF (20 ng/ml each, PeproTech) for 4 days *in vitro* (DIV). Neurospheres formed were then transferred onto mitomycin C-treated feeder layer, in Neurobasal medium with EGF and bFGF (20 ng/ml each) for neurosensory induction. From this point onwards, the cells were maintained at 37°C and 10% CO_2_. The medium was then changed to Neurobasal medium with EGF, bFGF, and a Rho kinase inhibitor Y27632 (25 *μ*M, Sigma-Aldrich) on the following day and again after 48 hours. Four hPSC lines have been shown to produce high proportions of neurosensory progenitors under these conditions [11, 15, 16].

### 2.4. Cochlear Explant Preparation

**Figure 1:**
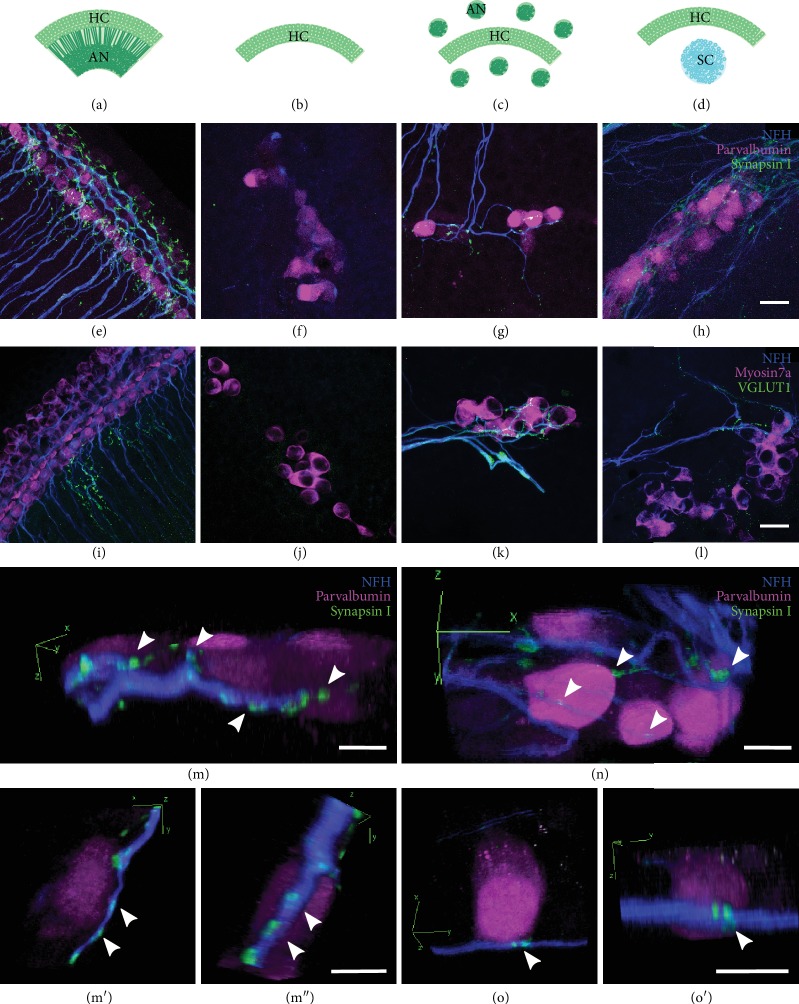
Stem cell-derived neurites innervated HCs within 12 days of coculture. Cocultures and explant controls were set up as follows: whole explant where HCs and ANs are kept intact (a), HC-only explant where ANs are dissected from HC rows (b), HC-AN coculture where HC rows and ANs are separated and cocultured (c), HC-SC coculture where HC rows are cocultured with stem cell-derived neurons (d). Whole explant controls were cultured for 1 day *in vitro* (DIV), whereas HC-only controls, HC-AN cocultures, and HC-SC cocultures were maintained for 12-14 DIV. Control cultures and cocultures were immunostained with antibodies for neurofilament for neurons (blue, (e)–(l)), parvalbumin for hair cells (magenta, (e)–(l)), and synaptic markers synapsin I (green, (e)–(h)) and VGLUT1 (green, (i)–(l)). Synapsin I was observed at the sites of innervation in whole explant control (e), HC-AN coculture (g), and HC-SC coculture (h), whereas it was absent in HC-only control (f). Similarly, VGLUT1 was observed at some sites of innervation in whole explant control (i), HC-AN coculture (k), and HC-SC coculture (l), whereas it was absent in HC-only control (j). Whole explant controls are cultured for 1 DIV and the explant maintains its ultrastructure. HCs in whole explant controls always appear smaller compared to cocultures and HC-only controls, since there is more loss of supporting cells and ultrastructure after weeks *in vitro*. (e)–(l) were all taken under oil immersion at the same magnification. (m, m′, m^″^) *x*/*y*/*z* reconstructions illustrate HC-AN innervation from three different directions (HC: magenta, parvalbumin; AN: blue, neurofilament; synaptic puncta: green, synapsin 1). (n) The *x*/*y*/*z* coordinates are reconstructed to illustrate SC-HC innervation and higher magnification images of SC-HC synapses is shown from two directions in (o) and (o′). Arrowheads in (m) through (o′) inclusive denote putative puncta between hair cells and either AN (m, m′, m^″^) or SC (n, o, o′). Scale bars (l) = 20 *μ*m, applies to (e)–(l). Scale bars in (m), (m^″^) (applies to (m′)), (n), and (o′) (applies to (o)) = 10 *μ*m.

Cochlear dissections were performed as previously described [11, 19]. Whole cochlear explants containing intact hair cell rows and attached auditory neurons (whole explants, Figure 1(a)) and hair cell- (HC-) only explants which were denervated and contained only hair cells (Figure 1(b)) were dissected from early postnatal rats aged between 1 and 3 days old (P1–P3). Auditory neurons (ANs) were further dissected by cutting the modiolus into smaller fragments of approximately 300 *μ*m^2^ in order to prepare HC-AN cocultures (Figure 1(c)). Small modiolar fragments were necessary in order to induce neurite outgrown from endogenous tissue [20]. All tissues were cultured on 0.4 *μ*m organotypic membranes (Millipore) in media described below.

### 2.5. Cochlear Nucleus Slice Preparation

The protocol for preparation of the cochlear nucleus (CN) slice was adapted from previously described rat brainstem slice culture protocols [21–24] and then combining these with protocols for embryonic mouse brain slice culture [25–27]. Briefly, P9-12 rat brain was dissected in cold artificial cerebrospinal fluid (ACSF; sodium chloride 125 mM, sodium bicarbonate 25 mM, potassium chloride 2.5 mM, magnesium chloride 1 mM, sodium phosphate monobasic 1.25 mM, and 0.45% glucose, oxygenated with carbogen gas (95% oxygen and 5% carbon dioxide) and made into a slushy by freezing at -20°C and mixing) and embedded in 3.5% SeaPlaque low melting temperature agarose (Lonza) in ACSF. Brainstem slices were cut using a vibratome. To obtain thin (200 *μ*m) slices, the embedded brain was kept at 2°C by placing it in a specimen bath filled with ACSF slushy, which was bubbled with carbogen gas. Brainstem slices containing CN were collected in ACSF slushy, and CN portions within brainstem slices were dissected. Anatomical confirmation of cochlear nucleus identity was confirmed using immunohistochemistry to neuronal marker NFH and synaptic markers VGLUT1 and synapsin I (Supplementary [Supplementary-material supplementary-material-1]).

### 2.6. Cocultures

Cocultures were set up by placing HC explants or CN slices with hPSC-derived neurons (as illustrated in Tables 1 and 2, respectively) at a distance of approximately 500 *μ*m apart on organotypic membranes. These cultures were maintained for 12-14 days in Neurobasal medium supplemented with brain-derived neurotrophic factor (BDNF; Chemicon) and neurotrophin-3 (NT3; Chemicon) with final concentrations of 10 ng/ml for each. For peripheral innervation assays, HC-SC cocultures were set up alongside whole cochlear explant controls, HC-only controls, and HC-AN control cocultures (Table 1). Whole explants were used as positive controls for immunochemistry under identical culture conditions to the treatments and cultured for 1 day to gain a measurement of “normal innervation” under the described culture conditions. Overnight culture was required in order to facilitate attachment of the hair cell explant to the organotypic membrane. Multiple AN samples were placed along HC sample in the HC-AN control group to increase the numbers of ANs to a density commensurate with the numbers of neurons in the SC spheres. These measurements were based upon the total numbers of cells within the stem cell spheres (~50,000 with approximately 80% of these being sensory neurons) and the numbers of neurons in the mammalian cochlear modiolus (~35-50,000 neurons *in situ,* although this number varies between species and may vary slightly between dissections). Since the whole modiolus does not grow well as a single piece of tissue, we cut this into several smaller explants and placed these around the HC explant. This allowed adequate access to the culture medium containing essential growth factors. For central innervation assays, CN-SC cocultures were set up alongside CN-only controls and SC-only controls (Table 2). Endogenous ANs were not cultured with endogenous CN slices in these assays as these two tissue types could not be differentiated without the use of genetically modified (GFP-positive) rats. For three-way innervation assays, HC-SC-CN cocultures were set up alongside HC-AN-CN control cocultures, under the same conditions described above.

### 2.7. Immunochemistry

All cultures were immunostained based on standard procedures which followed those previously described [10]. In brief, all cultures were fixed by immersing in 4% paraformaldehyde for 10 minutes. Whole cochlear explants were fixed after 1 day (controls), whereas all other cocultures were fixed at 12-14 DIV. Once fixed, the cultured cells were incubated in blocking solution (10% normal goat serum (Abacus ALS) or normal donkey serum (Millipore) and 0.1% TX-100 (Sigma-Aldrich) in phosphate-buffered saline) for 1 hour. The blocking solution was then replaced by a relevant combination of primary antibodies diluted in the blocking solution and incubated overnight at 4°C in a humidified chamber. The primary antibodies used were mouse anti-CtBP2 (1 : 400; BD Biosciences; 612044), mouse anti-human NFM (1 : 1000; Abnova; MAB5186), rabbit anti-Myosin VIIa (1 : 100, Sapphire Bioscience; 25-6790), chicken anti-NFH (1 : 800; Millipore; AB5539), chicken anti-NFM (1 : 2000; Millipore; AB5735), mouse anti-parvalbumin (1 : 1000; Sigma; P3088), mouse anti-synapsin I (1 : 1000; Synaptic Systems; 106001), rabbit anti-synapsin I (1 : 200; Invitrogen; A6642), mouse anti-VGLUT1 (1 : 1000; Millipore; MAB5502), rabbit anti-vesicular glutamate transporter 1 (VGLUT1; 1 : 3000; Sigma; V0389), and goat anti-parvalbumin (1 : 3000; Swant; PVG-214). Following the primary antibody incubation, the slides were washed 8 times, 15 minutes each, with the blocking solution. The slides were then incubated in a combination of appropriate secondary antibodies (Invitrogen and Jackson ImmunoResearch) diluted in PBS with 10% normal goat or donkey serum and 0.1% Tween 20 (Sigma-Aldrich) for 1.5 hours.

Following the incubation, the cultures were washed 3 times for 5 minutes each in PBS and the membranes mounted under coverslips using ProLong Gold mounting medium (Invitrogen). Samples were allowed to dry overnight at room temperature and coverslips sealed with nail varnish. Positive control experiments were performed on adult cochlear and brain sections, whilst negative controls were incubated in blocking solution without primary antibody.

### 2.8. Imaging and Quantification

Fluorescence images were captured using a Zeiss Axioplan upright microscope fitted with LSM510 Meta scanning laser system. Zen digital imaging software 2009 (Zeiss) was used to capture and process the images. Z-stack functions were used to capture appropriate sections of the cultures containing hair cells and neurites. Quantification of innervation in the peripheral innervation assay was performed using a Zeiss Axioplan 2 upright fluorescence microscope. Under 63x magnification, multichannel images of HCs, synaptic labelling, and neurites were taken and projected in an *x*, *y*, *z*, three-dimensional plane (Figures 1(m)–1(o), inclusive). The number of HCs innervated by direct contact with hPSC-derived neurites was counted. In addition, the numbers of hPSC-derived neurites within 100 *μ*m distance from HCs were counted (and this figure did not include those counted previously as touching the HCs). Neurite branching was not considered in this experimental model but could be in future using neural tracing. For hPSC-HC cocultures, we removed endogenous neurons in HC explant cultures, so we quantified all puncta as newly generated synaptic puncta. For hPSC-CN cocultures, we used human NFM antibody to distinguish hPSC-derived neurites from endogenous neurites and considered synaptic puncta adjacent to human NFM to be newly formed synaptic puncta. For HC-hPSC-CN cocultures, we identified neurites based upon morphology and location. Cocultures with ambiguous morphology were excluded from quantification. Quantitative data was analysed by the University of Melbourne's Statistical Consulting Centre, who employed independent samples *t*-tests of a minimum of 3 repeated coculture experiments using Minitab 17 statistical software. The standard error of the mean was reported with the mean as the measure of variability.

The number and volume of VGLUT1 puncta in central innervation assays were quantified using Analyze Particles function in ImageJ. To control for the slight differences in the amount of neurofilament within different parts of the CN slice, results were normalised to the overall size of neurofilament-positive area within the frame. To analyse statistical differences for means between CN-SC coculture and CN slice control, independent samples *t*-test (without assuming equal variance) was performed using Minitab 17 statistical software. Sample size used was *n* = 4 for CN-only controls, and *n* = 6 for CN-SC cocultures. The standard error of the mean (SEM) was reported with the mean as the measure of precision of the estimates of the means. Statistical analysis of data was performed in consultation with biostatisticians at the University of Melbourne.

For quantification of the number of CtBP2 puncta, z-stack multichannel images (CtBP2 and parvalbumin) were split into two channels. The parvalbumin z-stack image was flattened, and 10 HCs were outlined using the freehand selection tool (ImageJ). The selection was then restored onto the corresponding CtBP2 z-stack image, with all other areas being cleared. The number of CtBP2 puncta within the selected area was then objectively quantified using 3D Object Counter plugin (ImageJ). To analyse the statistical difference in numbers of CtBP2 puncta number between different treatment groups, independent samples *t*-test was performed using Minitab 17 statistical software: explant control 1 DIV, *n* = 5; HC control 1 DIV, *n* = 6; explant control 12 DIV, *n* = 7; and HC control 12 DIV, *n* = 7. Standard error of the mean was used as the measure of variability in the graph (Supplementary [Supplementary-material supplementary-material-1]).

## 3. Results

Human PSC lines were differentiated towards a neurosensory lineage as previously described [11, 15], supported by extensive molecular [16, 17] and physiological [16, 18] characterisation of these differentiated phenotypes. Differentiated progeny contained numerous AN-like features including action potential profiles akin to embryonic mammalian ANs [18] and showed temporal expression of key proteins and transcription factors such as Pax2, Sox2, NeuroD1, Brn3a, GATA3, Islet 1, parvalbumin, Trk B, TrkC, and VGLUT1 [15, 16].

### 3.1. Stem Cell-Derived Neurons Routinely Formed Synapsin I and VGLUT1-Positive Contacts with Postnatal Rat Cochlear HCs *In Vitro*

**Figure 2:**
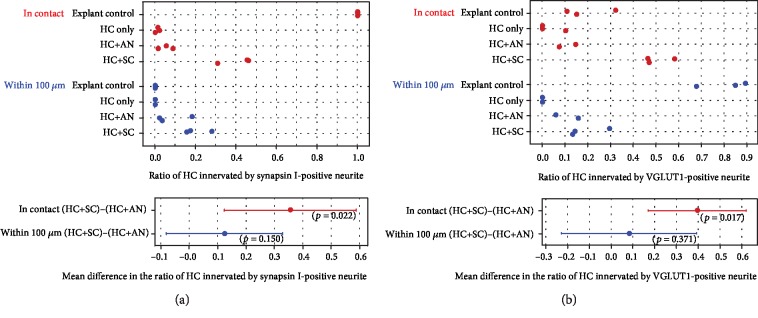
Quantification of stem cell-derived innervation of HCs within 12 days of coculture. The graphs depict the ratio of HCs innervated by synapsin I-positive neurites (a) and VGLUT1-positive neurites (b) in both control conditions and with SCs. The ratio is calculated as the observed innervation of HCs divided by the total numbers of HCs in the preparation. Innervation is shown in red (dots) and close proximity (within 100 *μ*m) is shown in blue (dots). Quantification of innervation for within the 100 *μ*m group did not include those puncta in contact with the HC. The ratio of HCs innervated by synapsin-positive neurites (a) and VGLUT1-positive neurites (b) was significantly higher (*p* < 0.05, independent samples *t*-test) in HC-SC cocultures compared with HC-AN cocultures. HC: hair cell; AN: auditory neurons; SC: differentiated stem cells.

Human PSC-derived sensory neurons (SC) were cocultured with auditory HC-only explants from P1-3 rats (Figure 1(d)) then fixed after 12 days *in vitro*. They were also cultured in the absence of any other cell types (Supplementary [Supplementary-material supplementary-material-1]). Three other conditions were tested in this assay: (1) whole explant positive control containing intact HCs and ANs at 1 DIV (Figure 1(a)); (2) HC-only negative controls at 12 DIV (Figure 1(b)); and (3) HC-AN endogenous controls containing the HC-only explant and dissected AN explants cultured together for 12 DIV (Figure 1(c)). To allow better access to the culture medium and to increase the survival of ANs in the culture, multiple AN explants were added per HC explant [20].

To visualise the sites of contact between hPSC-derived neurons and rat HCs *in vitro*, the cocultures and control cultures were first immunostained with parvalbumin (for HCs), neurofilament (NFH, for neurons and neurites), and synapsin I (for presynaptic terminals; Figures 1(e)–1(h) and 1(m), 1(m′), 1(m^″^)). In whole explant controls, the organised arrangement of HCs (one row of IHCs and 3 rows of OHCs) and radial bundles of AN neurites is maintained at 1 DIV (Figure 1(e)). Synapsin I immunoreactivity was observed at the sites of innervation as well as within the peripheral neurites. In HC-only control cultures after 12 days, the HCs showed a weak accumulation of neurofilament at the basal surface of the HCs (Figure 1(f)). No contamination of AN-derived neurites was observed in any of the HC-only controls. In HC-AN endogenous controls after 12 days, some ANs were observed to innervate HCs and synapsin I immunoreactivity was observed at the sites of innervation (Figures 1(g), 1(m), 1(m′), and 1(m^″^)). The arrangement of HCs at 12 days of coculture was less organised, as previously described [11]. In HC-SC cocultures after 12 days, neurites from stem cell-derived neurons were observed innervating HCs (Figures 1(h), 1(n), 1(o), and 1(o′)). The calibre of hPSC-derived neurites was noticeably finer than that of the explanted ANs in culture. These hPSC-derived neurites were positive for synapsin I at sites of innervation, as well as within close proximity to sites of innervation and within the neurospheres. SC-only spheres were also positive for synapsin I and VGLUT1 (Supplementary [Supplementary-material supplementary-material-1]). All HC-SC cocultures and HC-AN controls showed evidence of reinnervation, suggesting a capacity for both early postnatal and hPSC-derived neurons to reconnect with early postnatal cochlear HCs. We performed CtBP2:PSD-95 immunolabelling in an attempt to demonstrate polarized synapses in the described cultures but observed very low numbers of these. Whilst absolute numbers of CtBP2 puncta decrease with time in culture (Supplementary [Supplementary-material supplementary-material-1]), we suspect that the absence of these terminals is more likely an effect of the limited timeline for reinnervation.

Synaptogenesis was further investigated in the described cultures using VGLUT1 antibody (Figures 1(i)–1(l)). Cocultures and control cultures were established for 12 to 14 days and immunostained for HCs (Myosin VIIa), neurons (NFH), and synaptic vesicles (VGLUT1). In whole explant controls and endogenous AN explants, VGLUT1 immunostaining was observed within a proportion of peripheral neurites as well as at sites of innervation with HCs (Figure 1(i)). The punctate staining for VGLUT1 along the neurites was not present in all neurites within whole explants. In HC-only controls, negligible VGLUT1 immunoreactivity was observed (11 immunopositive HCs from a total of 383 HCs examined; Figure 1(j)). In HC-AN controls, all the neurites innervating the HCs were positive for VGLUT1 and new puncta were detected at sites of reinnervation with HCs (Figure 1(k)). In HC-SC cocultures, hPSC-derived neurites innervated the HCs with VGLUT1-positive neurites in a similar manner to that observed for endogenous AN cocultured with HCs alone (Figure 1(l)).

To quantify the extent of HC innervation by hPSC-derived neurons, the number of HCs innervated with synapsin I-positive neurites from hPSCs was compared with the number of total HCs in cultures (Figure 2(a)). The experiments involved a minimum of 3 repeats, and each point shown on the graph (Figure 2(a)) represents an average value calculated from at least two samples included in each condition. In whole explant controls (*n* = 5), all HCs (*n* = 205) were innervated by synapsin I-positive AN neurites. Residual synapsin I-positive terminals were observed in 1.15% ± 0.63% (SEM) of preparations from HC-only controls (*n* = 7; quantified from 344 HCs). In HC-AN controls (*n* = 3), 5.25% ± 2.1% of HCs were reinnervated by synapsin I-positive AN neurites (quantified from 503 HCs). An additional 7.92% ± 5.15% of HCs were not innervated but were in close proximity (within 100 *μ*m) to synapsin I-positive AN neurites. In HC-SC cocultures (*n* = 16), nearly half of the HCs were innervated by synapsin I-positive stem cell-derived neurons (40.82% ± 4.97%, quantified from 1824 HCs). Moreover, an additional 20.35% ± 3.89% of the HCs were in close proximity to these synapsin I-positive neurites. Innervation of HCs in HC-SC cocultures was routinely observed, and the ratio of innervated HCs was significantly higher in these cocultures in comparison to HC-AN cocultures (*t*-test: mean difference of 35.6, 95% confidence interval (CI) of 12.4 to 58.8, *p* = 0.022).

Using the same approach, HC innervation by VGLUT1-positive stem cell-derived neurites was quantified (Figure 2(b)). The portion of HCs innervated by VGLUT1-positive AN neurites in whole explant controls (*n* = 5) was 19.32% ± 6.57% (SEM), although this VGLUT1 innervation was well distributed since 80.67% ± 6.57% of total HCs had VGLUT1-positive AN neurites within close proximity to the HCs (quantified from 1440 HCs). Denervated HC-only controls (*n* = 3) had a small number of HCs positive for VGLUT1 (3.36% ± 3.36% SEM quantified from 383 HCs). HC-only control explants run in parallel to stem cell cocultures were entirely denervated, providing evidence that the NFM-positive processes in these cocultures arose from bona fide hPSC-derived neurons. In HC-AN controls (*n* = 3), 10.96% ± 3.61% of HCs were innervated by VGLUT1-positive AN neurites, and 10.82% ± 4.93% of the HCs were in close proximity to VGLUT1-positive AN neurites (within 100 *μ*m but not innervated, quantified from 662 HCs). In HC-SC cocultures (*n* = 11), significantly more HCs (54.13% ± 3.58%) were innervated by VGLUT1-positive stem cell-derived neurites. An additional 20.19% ± 4.86% of HCs were in close proximity to VGLUT1-positive hPSC-derived neurites (within 100 *μ*m but not innervated, quantified from 1663 HCs). The ratio of innervated HCs in HC-SC cocultures was calculated to be significantly higher compared to HC-AN controls (*t*-test: mean difference of 39.5, 95% CI of 16.9, 62.1, *p* = 0.017).

### 3.2. Stem Cell-Derived Neurons Innervated CN Slices with Synapsin I and VGLUT1-Positive Neurites within 14 Days *In Vitro*

**Figure 3:**
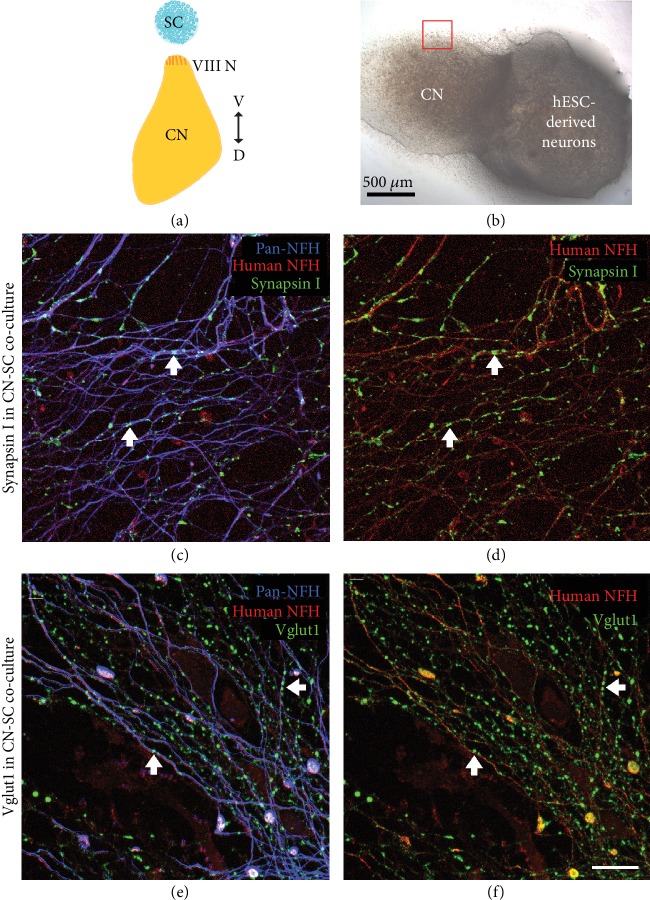
Stem cell-derived neurons innervated the edge of CN slices within 14 days. Stem cell-derived neurons were cocultured with rat CN slices with the VIII nerve (ventral side) facing towards them (a). Within 14 days of culture, stem cell-derived neurons extensively innervated the edge of CN slices in cocultures (b, red square). These stem cell-derived neurites (positive for human NFM (red) and NFH (blue)) (c–f) innervated the edge of the CN slices and were immunopositive for synaptic markers synapsin I (c, d) and VGLUT1 (e, f). Scale bar in (f) = 20 *μ*m, applies to all panels (c)–(f).

**Figure 4:**
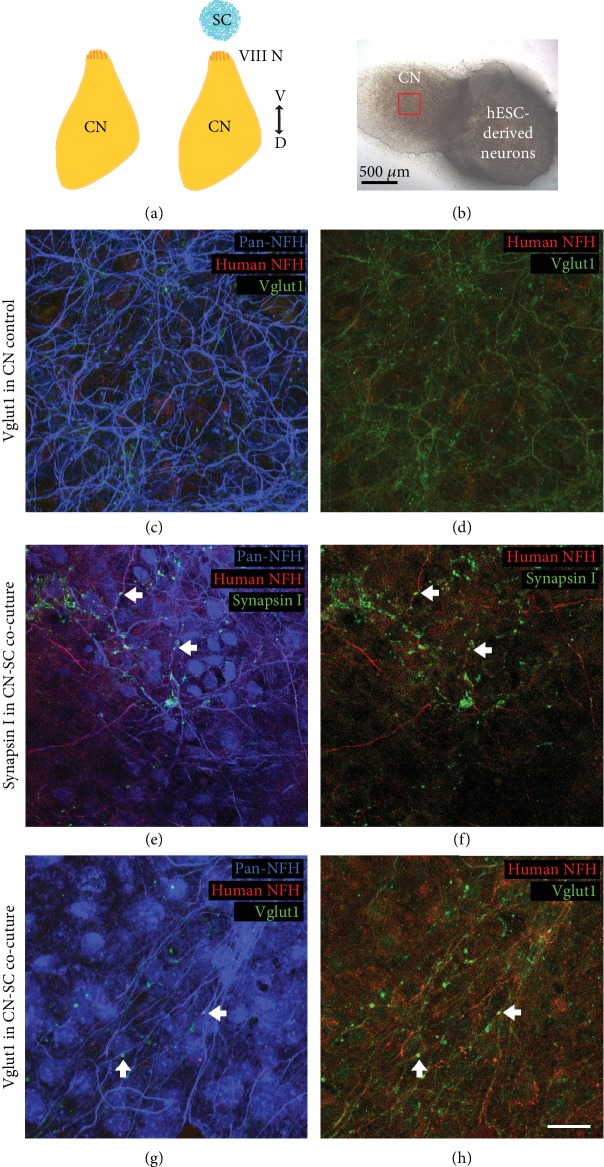
Stem cell-derived neurons innervated the centre of CN slices within 14 days. Cochlear nucleus (CN) slices were cultured with (right, a) or without (left, a) stem cell- (SC-) derived neurons for 14 days. After 14 days, both the CN-only (control) cultures and CN-SC cocultures were immunostained for neuronal (NFH, blue) (c, e, g), human neuronal (hNFM, red) (d, f, h), and the synaptic markers synapsin1 and VGLUT 1 (synapsin I: green (e, f) and VGLUT1: green (c, d, g, h)). In the CN-SC slice cocultures (b, red square), SC-derived neurites were observed innervating CN neurons within the centre of the slice (red, e–h). The stem cell-derived neurites were positive for both synapsin I (e, f) and VGLUT1 (g, h), similar to stem cell-derived neurites on the edge of CN slices. CN-only slice control (c, d) showed some VGLUT1 immunoreactivity but no hNFM immunoreactivity (g, h). Scale bar in (b) = 500 *μ*m; scale bar in (h) = 20 *μ*m, applies to all panels (c)–(h).

To investigate whether hPSC-derived neurons are capable of innervating central target cells in the CN, they were cocultured with CN slices on organotypic culture membranes for 14 DIV (Figures 3(a) and 3(b)). CN-SC cocultures were first immunostained for pan-neurofilament (NFH), human neurofilament (hNFM), and synaptic puncta (synapsin I; Figures 3(c)–3(f)). Following 14 days of culture, extensive innervation by hNFM-positive neurites was observed both around the edge of CN slices (Figures 3(c)–3(f)) and within the slices (Figures 4(e)–4(h)) by synapsin I colocalisation (Figures 3(d) and 3(f)). In addition to the immunoreactivity to hNFM antibody, the fine calibre of hPSC-derived neurites (which also normally grew straighter and within bundles) was easily distinguishable from the thicker endogenous neurites found in CN slices.

When these cocultures were immunostained for VGLUT1, VGLUT1-positive puncta were also observed within human NFM-positive neurites, both around the CN slice (Figures 3(e) and 3(f)) and within the slice (Figures 4(g) and 4(h)). CN-only controls did not show any innervation around the edge of the CN slice, and VGLUT1 immunoreactivity was observed in the neurites only within the CN slices (Figures 4(c) and 4(d)). The amount of neurofilament and VGLUT1 observed within the CN slices varied slightly between CN slices, potentially as a result of variable anatomical locations in each slice cultured.

**Figure 5:**
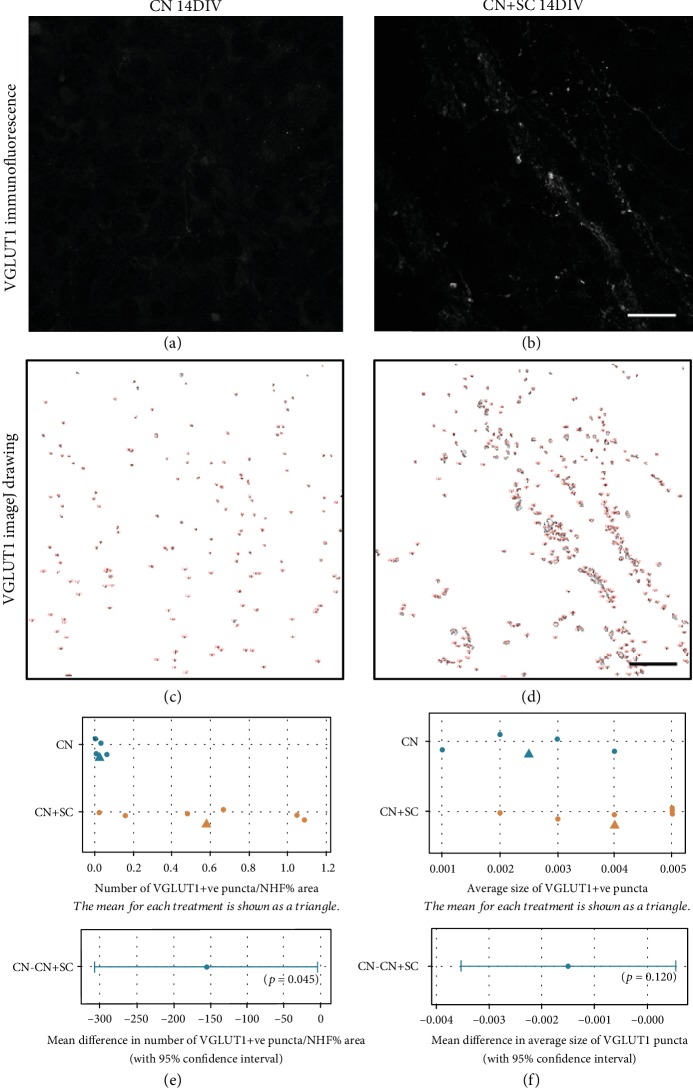
The number of VGLUT1-positive puncta was greater in CN-SC cocultures compared with CN slice controls. The number and size of VGLUT1 puncta in immunofluorescence images from CN-only controls (a) and CN-SC cocultures (b) at 14 DIV were analysed using ImageJ Analyze Particles function (c, d; ImageJ drawings of VGLUT1 puncta above a threshold). The number of VGLUT1-positive puncta (normalised to percentage of neurofilament-positive area in frame) was compared between CN-only controls and CN-SC cocultures. When independent two-sample *t*-tests were performed, the number of puncta was significantly higher compared with CN slice controls (e, *p* = 0.045, independent samples *t*-test with 95% confidence interval). Conversely, the average size of VGLUT1-positive puncta between the two groups did not differ significantly (ns) in these sample sizes (f, *t*-test: mean difference of -0.0015, 95% CI of -0.0035, 0.0005, *p* = 0.120). Scale bars in (b) and (d) = 20 *μ*m, applies to panels (a) and (c), respectively.

To quantify the degree of new (hPSC-derived) innervation in the CN slices, confocal images of CN controls and CN-SC cocultures immunostained for VGLUT1 and neurofilament were used. The VGLUT1 channel for CN-only control (Figure 5(a)) and CN-SC cocultures (Figure 5(b)) was converted into binary images and ImageJ's Analyze Particles function was used to select and count VGLUT1 puncta (Figures 5(c) and 5(d), respectively). To account for the difference in the amount of neurites and/or neurons in a given image, the percentage of neurofilament-positive area was used to normalise the number of VGLUT1-positive puncta in the image. The number of VGLUT1-positive puncta was significantly higher in CN-SC cocultures compared with CN controls (*t*-test: mean difference of -155.6, 95% CI -306.6, -4.6, *p* = 0.045, Figure 5(e)). Conversely, the difference in the size of puncta detected between CN-SC cocultures and CN-only controls was not significantly different (*t*-test: mean difference of -0.0015 and 95% CI -0.0035, 0.0005, *p* = 0.120, Figure 5(f)).

### 3.3. Growth of Endogenous Postnatal Auditory Neurons and Stem Cell-Derived Neurons in Three-Way Cocultures

**Figure 6:**
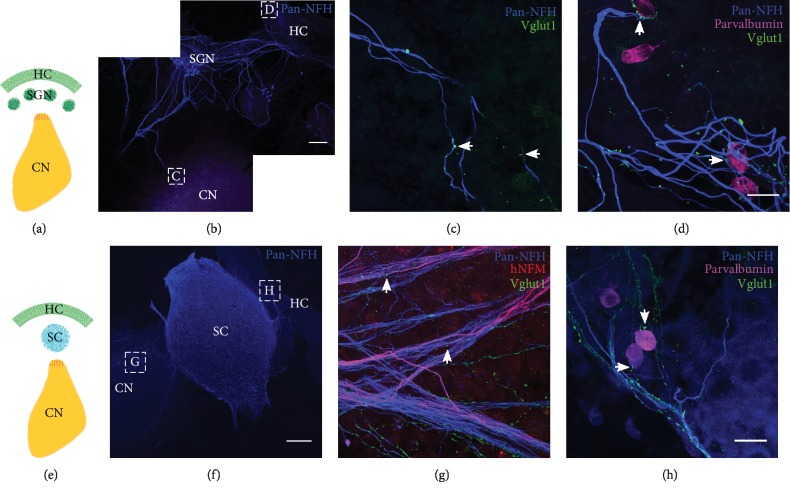
Stem cell-derived neurons and early postnatal ANs innervate both inner ear HCs and CN slice within 14 days. Both AN explants (a) and hPSC-derived neurons (SC, e) were cocultured with both HC-only explant and CN slice for 14 days. HC-AN-CN cocultures were stained for neurofilament (pan-NFH, blue), hair cells (parvalbumin, magenta), and VGLUT1 (green). Numerous processes of ANs extended towards both CN slice and HC explant (b). At higher magnification, ANs were observed to innervate the CN slice (c, arrows) and the HCs (d, arrows) with VGLUT1-positive neurites. HC-SC-CN cocultures were stained for neurofilament (pan-NFH, blue), hair cells (parvalbumin, magenta), human neurofilament (hNFM, red), and VGLUT1 (green). Fasciculating bundles of SC-derived neurons extended towards both CN slice and HC explant (f). At higher magnification, SC-derived neurites were observed to innervate the CN slice (g, arrows) and HCs (h, arrows) with VGLUT1-positive neurites. Scale bar in (b) and (f) = 200 *μ*m; scale bar in (d) and (h) = 20 *μ*m, applies to panels (c) and (g), respectively.

To investigate whether endogenous ANs or hPSC-derived neurons could grow toward peripheral (HC) or central (CN) target cell populations in the same coculture, a further *in vitro* coculture model was established. In these experiments, either auditory neurons (Figures 6(a)–6(d)) or hPSC-derived neurons (Figures 6(e)–6(h)) were tested for their ability to innervate both cochlear HCs and CN neurons after 14 days *in vitro*. Optimal cell culture conditions for the simultaneous growth of these three cell types were assessed independently prior to experimentation, using live/dead assays at several time points under different defined media conditions (Supplementary [Supplementary-material supplementary-material-1]).

Immunostained cocultures (Figures 6(b)–6(d) and 6(f)–6(h)) illustrate all neurons (Pan-NFH; blue), synaptic contacts (VGLUT1; green), and HCs (parvalbumin; magenta). In endogenous cocultures, ANs were observed to grow their neural processes towards HCs (Figures 6(b) and 6(d)), towards other AN explants on the membrane (Figure 6(b)) and towards the CN slice (Figures 6(b) and 6(c)). VGLUT1-positive puncta were observed within endogenous AN neurites (Figures 6(c) and 6(d)) also at the sites of peripheral and central innervation (Figures 6(c) and 6(d)). Semiquantitative analyses revealed that both the HCs and CN slice were innervated by endogenous postnatal auditory neurons in at least 3 of 17 cocultures (from triplicate experimentation). This number was calculated from clear cases where both the HCs and the CN targets were definitively innervated by ANs. In cocultures where hPSC-derived neurons were cocultured with both HCs and CN slice, stem cells were observed to innervate both the HCs and the CN neurons in 17 of 21 cases (from triplicate experimentation). Greater numbers of hPSC-derived neurites were observed in these cocultures and displayed noticeably finer calibre neurites (Figures 6(g) and 6(h)) in comparison to those derived from endogenous ANs in cocultures (Figures 6(c) and 6(d)). VGLUT1 labelling was observed within hPSC-derived neurites that had grown into the CN slice (Figure 5(g)), and these were distinguished from endogenous CN neurites by hNFM staining (Figure 6(g)). VGLUT1-positive hPSC-derived neurites were also observed to contact the HCs (parvalbumin, Figure 6(h)) within the coculture. In a small number of cocultures, neurites from the CN slices extended directly towards HCs in the explants.

## 4. Discussion

Cocultures offer an alternative way to study cellular interactions in a microenvironment that is easily controlled and adjusted and at a fraction of the cost of *in vivo* experimentation. As a result, *in vitro* assays are understandably appealing for proof-of-concept and/or screening experimentation prior to pursuing *in vivo* alternatives. One drawback of *in vitro* models is that they do not confer an *in vivo* niche and thus may have a limited ability to provide such physiological conditions such as spontaneous activity or the precise biological concentrations and/or combinations of diffusible growth and guidance cues. Importantly, organotypic culture may overcome some of these drawbacks by providing the ability to culture slice and explant tissue (rather than dissociated cells) and therefore provide more similarity to the *in vivo* niche than dissociated cell cultures grown on submerged coverslips. In the present study, we used a range of organotypic cocultures to measure the extent to which hPSC-derived sensory neurons could make new synapses on inner ear HCs, neurons in CN slice or, HCs and CN slice neurons in the same culture. Extensive quantification and statistical analyses were performed to provide a more comprehensive picture of the frequency with which new synapses were observed between stem cells and auditory target tissues. These were then compared to endogenous controls grown under identical conditions.

In the present study, we observed stem cell-derived neurons contacting early postnatal cochlear hair cells within 12 days *in vitro* and these sites of contact contained puncta immunopositive for the presynaptic vesicles synapsin I and VGLUT1. These new synaptic connections morphologically resembled those present within endogenous tissue controls, including those auditory neural processes contained within the whole explant and also within explanted auditory neurons (Figure 1). Regardless of whether neural processes were stem cell-derived or endogenous, they contained presynaptic puncta for synapsin I and VGLUT 1 both within their processes and at the site of contacts with the hair cells (Figure 1, Supplementary [Supplementary-material supplementary-material-1]). The presence of synapsin in neurons at sites of innervation with HCs has been observed previously in studies coculturing mouse organ of Corti with mouse auditory neurons [28], mouse organ of Corti with mouse stem cell-derived neurons [8, 9], mouse organ of Corti with human stem cell-derived neurons [7], and rat organ of Corti with hPSC-derived neurons [10, 11]. In the latter study, preferential innervation of inner or outer hair cells was investigated [10]. These data lend further support to the abovementioned observations using human stem cell-derived neurons but with additional quantitative analyses using endogenous controls included for comparison (Figure 2). Importantly, the average percentage of HCs innervated by endogenous ANs was calculated as 5-11%, figures which are consistent with those reported in early postnatal mice (4-8%; [29]).

Previously, stem cell-derived neural precursors were shown to grow toward a CN slice and make SV2-positive terminals as they reached the CN slice [12, 13]. The current study demonstrates for the first time that hPSC-derived neurites are capable of penetrating growth into the CN slice *in vitro* (Figures 3 and 4). This is an important new finding, given that the success of future stem cell therapy in the inner ear requires targeted growth into the CN. As was observed in the HC-SC cocultures described in the present study, we also observed the expression of both synapsin I and VGLUT1 within stem cell-derived neurites in coculture with the CN slice. The expression of both synapsin1 and VGLUT1 was observed within hNF-positive neurites both at points of contact with endogenous CN neurons and also more diffusely within the neurite. These observations are consistent with recent studies culturing mouse stem cell-derived neurons with dissociated CN [30, 31]. When quantified, there was significantly greater innervation observed in cocultures of stem cells with CN slices, as determined by numbers of VGLUT1 puncta (Figure 5). Puncta size between CN-SC and endogenous CN cultures was not significantly different, further supporting anatomical similarities between hPSC-derived and endogenous neurons. In addition, the specific colocalisation of stem cell-derived VGLUT1 puncta around endogenous CN neuron cell bodies (Figure 4(f)) provides further evidence of their potential to make new contacts with neurons within the CN, a key finding for *in vivo* translation of these data.

Finally, we compared the ability of hPSC-derived neurons and endogenous ANs to make new synaptic contacts on HCs and CN tissues in a three-way coculture model (Figure 6). The described model was developed in an attempt to replicate the *in vivo* scenario, but with lower costs and as a proof-of-concept from which to proceed with *in vivo* application of stem cells to the inner ear. We report that both hPSC-derived neurons and endogenous ANs are capable of growth towards inner ear HCs and CN slice in the described organotypic coculture model after two weeks *in vitro*. Both hPSC-derived neurons and endogenous ANs expressed VGLUT1 within their neurites and at sites of innervation with HCs and CN neurons. Consistent with unidirectional assays, hPSC-derived neurons were capable of synaptogenesis in the described system. Further experimentation using reporter lines is required to interrogate whether single hPSC-derived neurons (or ANs) can reinnervate these target tissues simultaneously and may be improved by incorporating small numbers of endogenous ANs (as would likely be the scenario *in vivo*). In addition, incorporating markers of proliferation (for example KI-67) would help in determining the proliferative capacity of these neurospheres after five weeks of differentiation.

Collectively, the present study describes the development of a new auditory coculture model which facilitates further investigations of simultaneous, bidirectional growth of stem cells towards target cells and tissue in the inner ear and brainstem. The model is the first to quantify the frequency of human stem cell-derived innervation of peripheral and central auditory targets and compare this to relevant endogenous tissue controls. The described assays are useful for proof-of-concept studies, to validate and/or interrogate the usefulness of a variety of stem cell types prior to their *in vivo* application.

## Figures and Tables

**Table 1:** Summary of experimental groups for peripheral innervation cocultures.

	Whole explant control	HC-only control	AN/HC control	SC/HC coculture
Preparation	Whole cochlear explant where HCs and ANs are kept intact	HC-only explant where ANs are dissected from HCs	HC rows and ANs are separated and cocultured	HCs and SC-derived neurons: hair cell rows are cocultured with SC-derived neural progenitors
Culture period	1 DIV	12-14 DIV	12-14 DIV	12-14 DIV
Images	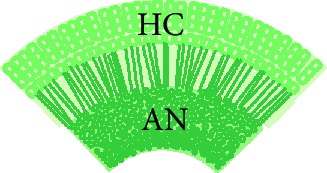	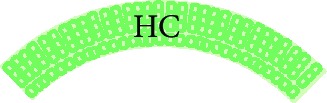	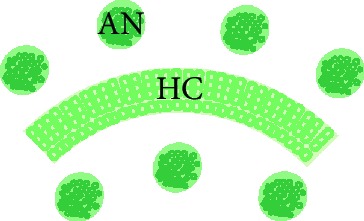	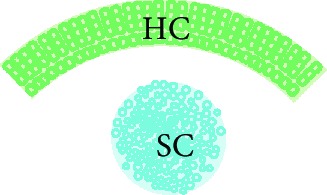
Controls for	Positive control for synapses between HCs and endogenous ANs	Negative control for synapses	For comparison between SC-derived neurons and endogenous ANs in reinnervation	Experimental group

HC: hair cell; AN: auditory neuron; SC: stem cell; DIV: days *in vitro*.

**Table 2:** Summary of experimental groups for central innervation cocultures.

	CN control	SC control	SC/CN coculture
Preparation	Cochlear nucleus slice (200 *μ*m thick)	Human PSC-derived neurons cultured on their own	Human PSC-derived neurons cultured with cochlear nucleus slice
Culture period	1 or 14 DIV	14 DIV	14 DIV
Image	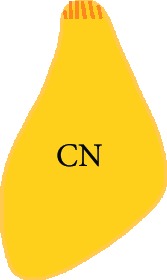	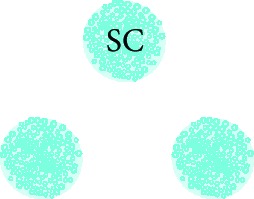	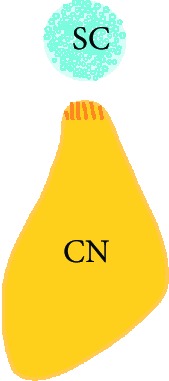
Controls for	The presence of VGLUT1 in cultured CN slice without addition of hPSC	The presence of VGLUT1 in cultured hPSC without CN	The specificity of hPSC-derived neural innervation

CN: cochlear nucleus; SC: stem cell; DIV: days *in vitro.*

## Data Availability

Data is recorded in laboratory notebooks and stored securely in the laboratory. Electronic imaging files are stored on University, Password-protected servers.

## Supplementary Materials

Supplementary Materials Supplementary Figure 1: anatomical characterisation of P12 cochlear nucleus and VIII nerve slice. Supplementary Figure 2: numbers of CtBP2 puncta decrease in culture over time. Supplementary Figure 3: expression of synaptic markers in SC-only cultures. Supplementary Figure 4: cochlear nucleus slice cell viability assays.
