# Supplementary material for: Organotypic Cocultures of Human Pluripotent Stem Cell Derived-Neurons with Mammalian Inner Ear Hair Cells and Cochlear Nucleus Slices
Source: Stem Cells Int. 2019 Nov 20;2019:8419493. doi: 10.1155/2019/8419493 (PMC6886346; doi:10.1155/2019/8419493)
Supplement: Supplementary Materials — Supplementary Figure 1: anatomical characterisation of P12 cochlear nucleus and VIII nerve slice. Supplementary Figure 2: numbers of CtBP2 puncta decrease in culture over time. Supplementary Figure 3: expression of synaptic markers in SC-only cultures. Supplementary Figure 4: cochlear nucleus slice cell viability assays. [file 8419493.f1.pdf]

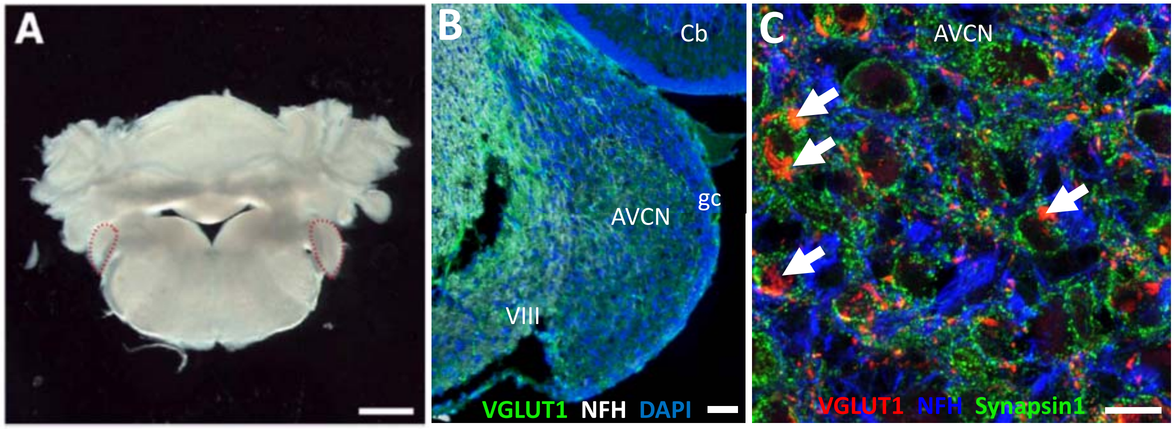

Supplementary Figure 1: Anatomical characterisation of P12 cochlear nucleus and VIII nerve slice. Cochlear nucleus slices containing the VIII nerve were obtained from post-natal day 12 (P12) rats (A; circled). Parasagittal sections containing the cochlear nucleus and VIII nerve (B) were selected and used for co-cultures. Typical cell populations were observed in the anteroventral cochlear nucleus (AVCN) using immunohistochemistry for neurofilament (NFH, blue B) and VGLUT1 (green, B). These characteristic cell morphologies included putative bushy cells in the core of the nucleus and much smaller granule cells (gc) along the lateral border of the AVCN, as described by others [32]. The VIII nerve (VIII) was located medial to the AVCN and the cerebellum (Cb) superior to the cochlear nuclei. Large VGLUT1-positive terminals (red, arrows C) were observed around putative bushy cells in the AVCN, whilst pre-synaptic terminals (green, C) were indicated by synapsin 1 labelling. Neurons and processes immunostained with neurofilament [NF; white (B) and blue (C)]. VIII: vestibulocohlear nerve; gc: granular cell layer. Scale bars A= 2.5 mm; B=250 µm; C=20 µm .

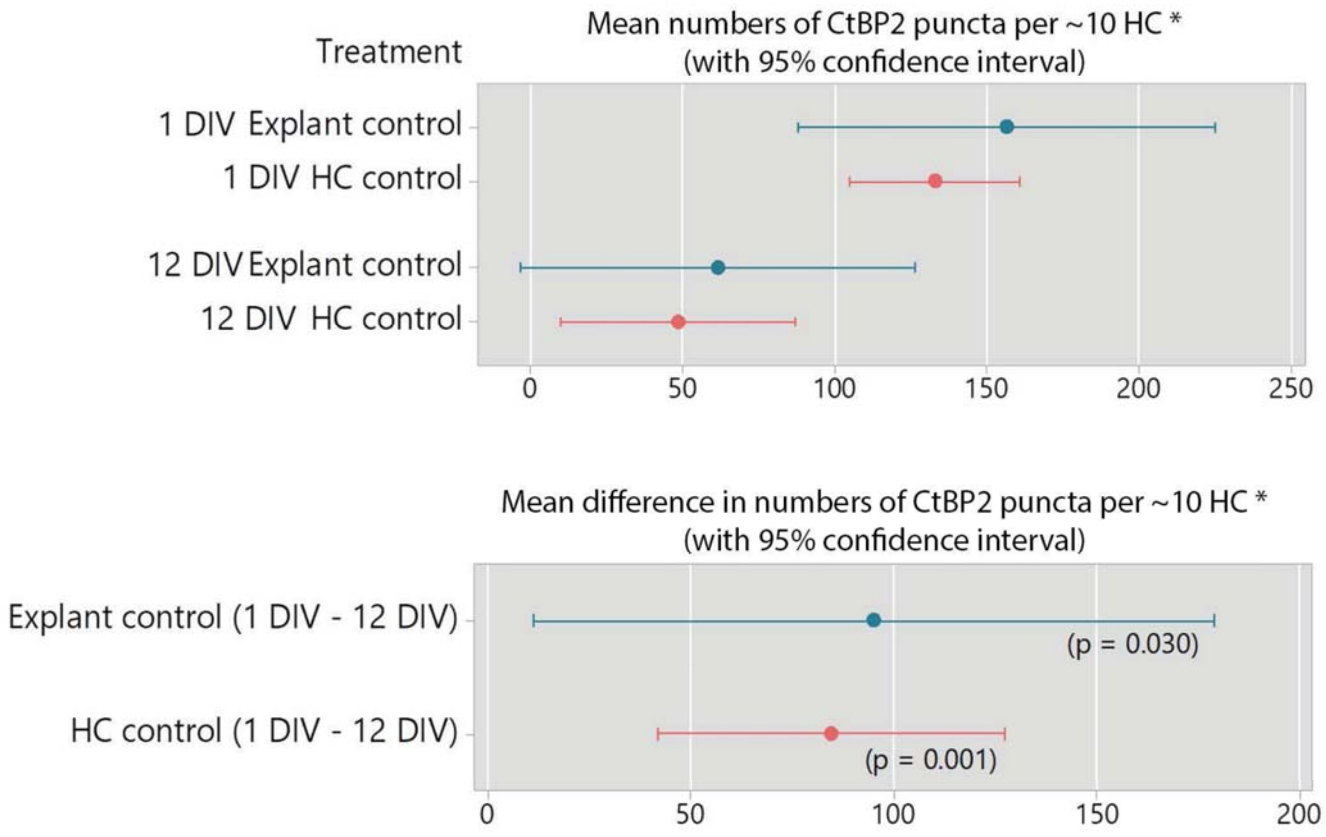

Supplementary Figure 2: Numbers of CtBP2 puncta decrease in culture over time. Antibody labelling was used to quantify the change in number of CtBP2 puncta in HCs over time in culture. At least 3 samples from each of the following treatments were used: whole explant at 1 DIV, HC-only explant at 1 DIV, whole explant at 12 DIV and HC-only explant at 12 DIV. *The numbers of CtBP2 within a parvalbumin-positive area equivalent to 10 HCs was counted using 3D object counter (ImageJ). For both whole explant control and HC only control, the number of CtBP2 puncta decreased significantly over time in culture ($p<0.05$, independent t-test with 95% confidence interval).

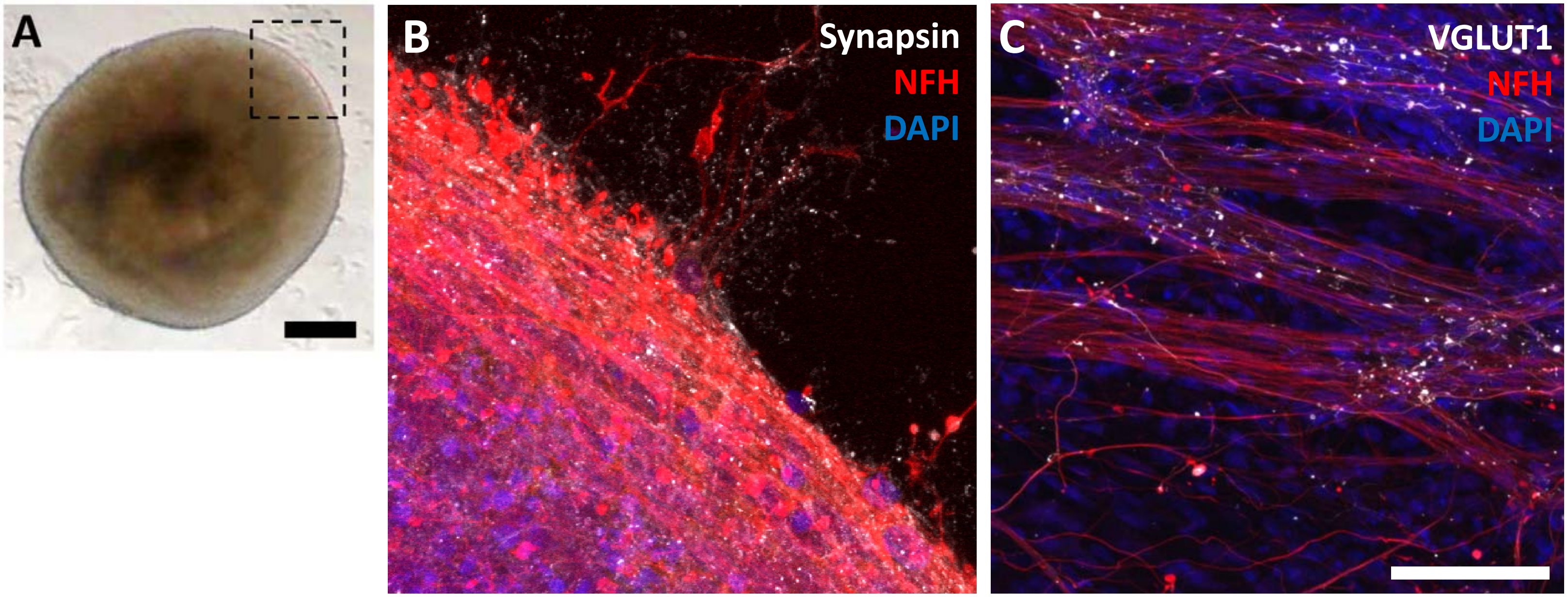

Supplementary Figure 3: Expression of synaptic markers in SC-only cultures. Human PSC neurospheres were grown on organotypic membranes in the absence of HC explants (A). Diffuse synapsin 1 expression was observed throughout neurospheres (white puncta, B) as was VGLUT1 (white, C). The volume of cells within the neurospheres along with their irregular spherical shape presented significant challenges for confocal imaging of synaptic puncta, thus the edges of the spheres were imaged to demonstrate the presence of puncta within stem cell-derived neurites. Scale bars A= 150 µm; C (applies to B) = 40 µm.

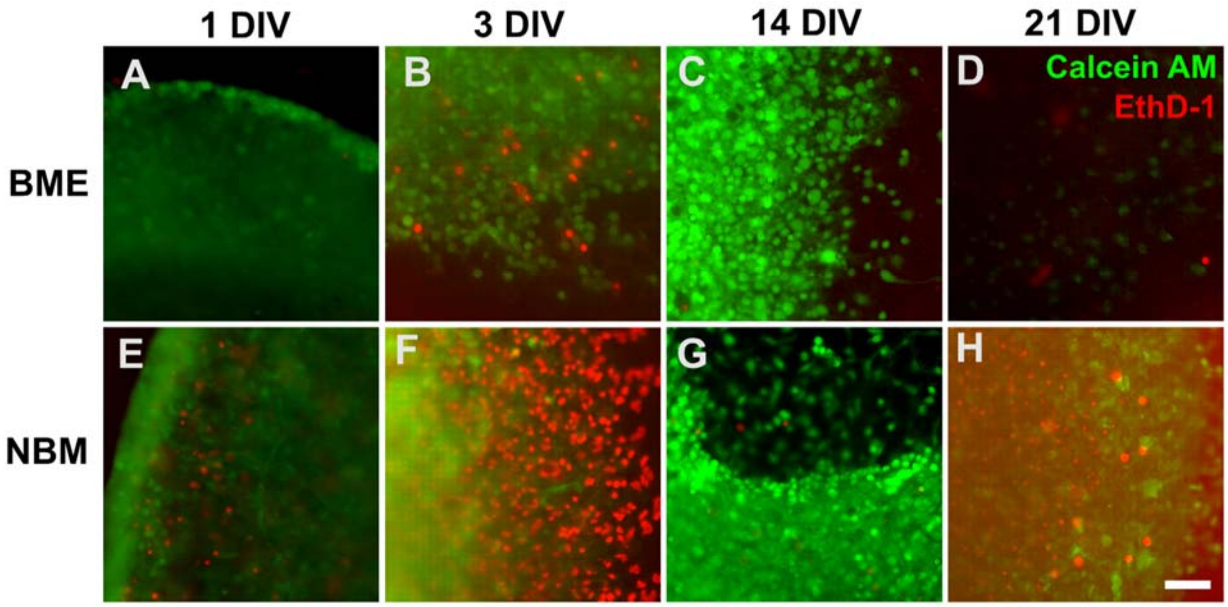

I

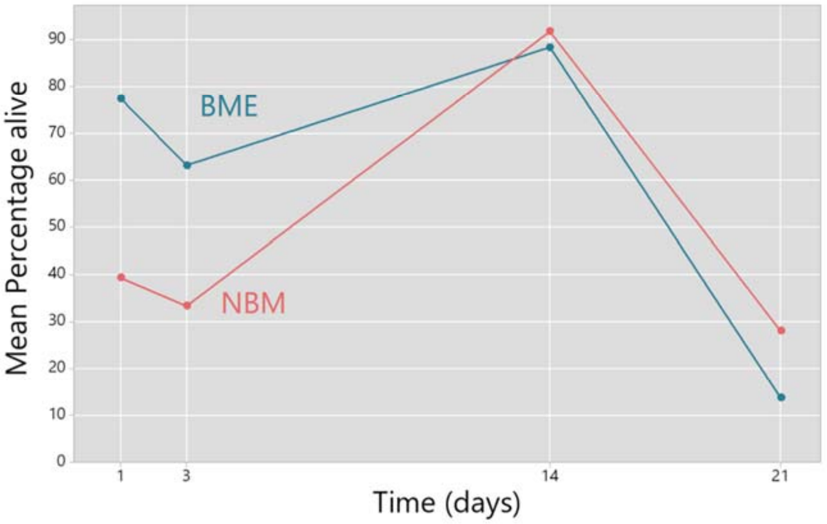

J

Time	Mean percentage alive NBM	BME	Mean difference NBM - BME	95% CI	*P*-value
1	39.3	77.4	-38.1	-62.0, -14.2	0.003
3	33.3	63.10	-29.8	-60.6, 1.1	0.058
14	91.73	88.31	3.42	-16.10, 22.94	0.719
21	28.0	13.7	14.3	-19.5, 48.1	0.388

Supplementary Figure 4: Cochlear nucleus slice cell viability assays. Cochlear nucleus slices were obtained from postnatal day 9-12 rats and cultured on organotypic membranes in two types of media (Neurobasal: NBM or Basal Medium Eagle: BME). Live-dead assays were performed at 1 (A, E) 3 (B, F), 14 (C, G) and 21 (D, H) days in culture. Live cells were stained green with Calcein AM, and dead cells were stained red with EthD-1 (A-H). There are some dead (or dying) cells in CN slices cultured in both media. At 14 DIV, the slices in both media have dense packs of viable CN cells. Scale bar = 50 µm (H), applies to all panels A-H. The intensity of immunofluorescence was measured using ImageJ to calculate the ratio of viable cells to dead cells (I). Mean peak cell viability was measured at 14 days in culture, the end point for the described co-cultures. Absolute values provided in (J) for comparison.
